# Predicting pathogenicity behavior in *Escherichia coli* population through a state dependent model and TRS profiling

**DOI:** 10.1371/journal.pcbi.1005931

**Published:** 2018-01-31

**Authors:** Krzysztof Bartoszek, Marta Majchrzak, Sebastian Sakowski, Anna B. Kubiak-Szeligowska, Ingemar Kaj, Pawel Parniewski

**Affiliations:** 1 Department of Mathematics, Uppsala University, Uppsala, Sweden; 2 Institute of Medical Biology, Polish Academy of Sciences, Lodz, Poland; 3 Faculty of Mathematics and Computer Science, University of Lodz, Lodz, Poland; University of California Irvine, UNITED STATES

## Abstract

The Binary State Speciation and Extinction (BiSSE) model is a branching process based model that allows the diversification rates to be controlled by a binary trait. We develop a general approach, based on the BiSSE model, for predicting pathogenicity in bacterial populations from microsatellites profiling data. A comprehensive approach for predicting pathogenicity in *E*. *coli* populations is proposed using the state-dependent branching process model combined with microsatellites TRS-PCR profiling. Additionally, we have evaluated the possibility of using the BiSSE model for estimating parameters from genetic data. We analyzed a real dataset (from 251 *E*. *coli* strains) and confirmed previous biological observations demonstrating a prevalence of some virulence traits in specific bacterial sub-groups. The method may be used to predict pathogenicity of other bacterial taxa.

## Author summary

An important challenge in Computational Biology is the analysis of genetic molecular data through sophisticated computer science and mathematical methods that are implemented by interdisciplinary research groups. The resulting comprehensive approach, based on the BiSSE model and microsatellites profiling (TRS-PCR), can be used to predict pathogenicity behavior in bacterial taxa. As proof of concept, we applied the procedure to real clinical data sets of genetic information obtained from a unique collection of bacterial populations (251 strains). Our results showed that a state-dependent model was able to predict pathogenicity behavior of *E*. *coli* population. Furthermore, we confirmed previous biological observations indicating a prevalence of some virulence genetic traits in bacteria.

## Introduction

The diverse species of *E*. *coli* display a large repertoire of genetic traits—pathogenicity factors, allowing the colonization of the human host. Depending on the occupied niche, individual virulence factors (VFs) are favored, allowing the survival of the pathogen. However, pathogenicity factors are maintained only when they favor the development of the pathogen, during the colonization of the host [1]. Otherwise they are eliminated when not required, as they are costly traits, or when their presence (expression) promotes detection by the host’s immune system [2,3].

Microsatellites, stretches of DNA consisting of repeated short segments of nucleotides (sequence motifs) are commonly found in bacterial genomes. A special class of microsatellites, that of trinucleotide repeat sequences (TRS motifs), is genetically unstable and this instability depends mainly on the length and number of copies of the repeated motif [4,5]. In the case of bacterial genomes a TRS rarely exceeds 10 copies and is therefore relatively stably transmitted in subsequent generations. The number of such loci (with the number of repetitions n ≥ 3) varies depending on the species of the microorganism and is, for example 1667 for *S*. *aureus* JH1, 2568 for *E*. *coli* CFT073 and 4201 in the case of *M*. *tuberculosis*. Amplification of DNA regions located between the TRS motifs allows one to obtain band patterns specific to the genus, species or a bacterial strain [6–8]. In the case of *E*. *coli*, CGG- and / or GTG-PCR patterns are correlated with their phylogenetic membership and also group strains having similar sets of VFs [9,10]. Therefore, the question is whether the observed phenomenon of clustering is only a reflection of the genetic *status quo*, or can it also be helpful in predicting directions of pathogenicity development in the *E*. *coli* population. Such a hypothesis was verified by employing the binary-state speciation and extinction model—BiSSE [11] with an appropriate probabilistic interpretation (see S1 Appendix).

BiSSE is a theoretical model, which was introduced into the phylogenetic community by Maddison et al. [11]. Apart from special subcases, see e.g. [12], the likelihood is not analytically tractable but can be obtained numerically by solving an ODE system (as in the diversitree R package [13–15]). Since its introduction a number of generalizations have been implemented such as quantitative state speciation and extinction (QuaSSE, [14]) where the speciation and extinction rates depend on an arbitrary (even continuous) suite of traits, or Hidden State Speciation and Extinction model (HiSSE, [16]). Even though the likelihood function is not analytically tractable one can deduce large sample properties of the model by studying branching processes on generalized state spaces. In particular Janson [17] provides results characterizing the limit behavior (almost sure convergence and central limit theorems).

In this paper we apply the BiSSE model to estimate parameters from a collection of 251 strains from the clinical isolates of *E*. *coli*. We present an application of microsatellites, specifically TRS microsatellites, as pathogenicity markers and we analyze *E*. *coli* strains using the BiSSE model.

## Methods

### *E*. *coli* strains used in this study and virulence factors characterization

A collection of 128 clinical *E*. *coli* strains (set U) was gathered between June 2005 and September 2006 from the urine of patients in various wards of the Military Teaching Hospital No. 2, Medical University of Lodz, Poland. The second collection (set K) composed of 123 isolated from children with diarrhea in the Lodz region (Poland) and were obtained from the Medical Laboratory SYNEVO in Lodz, Poland. Isolates were collected from January 2009 to May 2010. Genomic DNA isolation and purification was performed with the use of a GenElute Bacterial Genomic DNA Kit (Sigma-Aldrich, St. Louis, MO). The quantity and purity of each genomic DNA sample was determined spectrophotometrically at 260 nm (BioPhotometer, Eppendorf, Germany). The DNA samples were diluted to 20 ng/μl and then used. The possession of virulence genes, typical for uropathogenic (UPEC) and intestinal *E*. *coli* (IPEC) was determined by multiplex-PCR, according to procedures described elsewhere [9,10,18–22]. Detailed characteristics of the collection of strains are presented in Table 1 and Table 2.

**Table 1:** Number of VF features and their function in the K and U populations. K, strains isolated from children with diarrhea; U, strains isolated from patients with urinary tract infections. (grey zone–VFs underrepresented, not included for prediction).

Virulence factor	Function	Number of strains	
		K	U
*astA*	heat-stable enterotoxin 1	7	20
*cnf1*	cytotoxic necrotizing factor 1	3	38
*fimG*	fimbrial protein FimG	120	115
*fyuA*	pesticin/yersiniabactin receptor protein	74	78
*hly1*	alpha-hemolysin	5	40
*iroN*	IroN protein, siderophore receptor	36	84
*iutA*	ferric aerobactin receptor precusor IutA	77	62
*papC*	fimbrial protein	33	45
*sat*	secreted autotranspoter toxin	46	15
*sfa*	S fimbriae major subunit SfaA	2	43
*tsh*	temperature sensitive hemagglutinin	8	7
*usp*	uropathogen-specific protein, bacteriocin-like genotoxin	2	40
*escV*	Type III secretion system major export apparatus protein	14	0
*stx1*	shiga-like toxin I	1	0
*stx2*	shiga-like toxin II	1	0
*pic*	Pic serine protease precursor, autotransporter	2	3
*aggR*	AraC homolgous regulator of AAF/I	1	1

**Table 2:** The virulence factors characteristics of *E*. *coli* strains used in this work. (grey zone–VFs underrepresented, not included for prediction).

VF strain	*papC*	*sfa*	*cnf1*	*usp*	*hly1*	*fimG*	*astA*	*fyuA*	*iutA*	*iroN*	*sat*	*tsh*	*escV*	*stx1*	*stx2*	*pic*	*aggR*
**K 001**	1					1		1	1	1							
**K 002**	1					1											
**K 003**	1					1	1				1						
**K 004**						1		1		1	1						
**K 005**	1					1		1		1							
**K 006**						1		1	1	1	1						
**K 007**						1											
**K 008**						1		1	1				1				
**K 009**						1		1	1			1					
**K 010**						1			1								
**K 011**						1		1	1	1							
**K 012**						1		1	1				1	1			
**K 013**						1		1	1	1		1					
**K 014**	1	1	1	1	1	1		1			1						
**K 015**						1	1		1								
**K 016**						1					1						
**K 017**	1					1		1	1	1							
**K 018**	1					1			1								
**K 019**						1		1	1	1	1						
**K 020**						1					1						
**K 021**	1					1		1	1								
**K 022**						1					1						
**K 023**	1					1		1	1	1							
**K 024**						1		1	1		1						
**K 025**	1		1		1	1		1	1								
**K 026**						1		1	1	1		1					
**K 027**						1			1								
**K 028**	1					1		1									
**K 029**						1			1				1				
**K 030**						1					1						
**K 031**						1		1	1	1		1					
**K 032**						1							1				
**K 033**						1											
**K 034**						1		1		1							
**K 035**						1		1		1							
**K 036**	1					1		1	1	1							
**K 037**																	
**K 038**						1		1	1		1						
**K 039**						1		1	1		1						
**K 040**						1			1		1						
**K 041**	1					1		1	1	1	1						
**K 042**						1		1	1		1						
**K 043**	1							1	1	1							
**K 044**						1		1									
**K 046**						1							1		1		
**K 048**						1											
**K 049**	1					1		1	1	1							
**K 051**	1				1	1	1		1		1					1	
**K 052**	1					1		1	1	1							
**K 053**						1											
**K 055**						1		1	1		1						
**K 057**						1											
**K 059**	1					1		1	1	1							
**K 060**	1					1		1	1								
**K 061**						1		1	1		1						
**K 062**	1	1	1	1	1	1		1	1	1							
**K 063**						1			1		1						
**K 064**						1		1									
**K 065**						1			1		1						
**K 066**						1					1						
**K 067**						1		1	1		1						
**K 071**						1		1									
**K 072**	1					1		1	1		1						
**K 073**						1		1	1	1		1					
**K 074**	1					1		1	1	1							
**K 075**						1		1	1	1		1					
**K 076**						1		1	1		1						
**K 077**						1		1	1		1						
**K 078**	1					1		1	1	1							
**K 079**						1			1		1						
**K 080**						1		1	1		1						
**K 081**						1			1		1						
**K 082**						1		1	1	1		1					
**K 083**						1			1		1						
**K 084**						1		1	1		1						
**K 085**						1		1	1		1						
**K 086**						1			1		1						
**K 087**						1			1		1						
**K 089**						1			1		1						
**K 090**						1		1	1		1						
**K 091**	1					1		1	1		1						
**K 093**						1		1									
**K 094**	1					1		1	1	1							
**K 095**						1			1		1						
**K 096**						1		1									
**K 097**						1		1			1						
**K 098**						1											
**K 099**	1					1		1	1	1							
**K 100**						1											
**K 102**						1											
**K 103**						1											
**K 104**						1			1		1						
**K 106**						1							1				
**K 108**						1	1	1		1							
**K 110**						1		1	1		1						
**K 111**						1			1		1						
**K 112**						1		1	1		1						
**K 113**						1			1		1						
**K 114**						1		1	1		1						
**K 115**	1					1		1	1	1							
**K 116**						1		1	1				1				
**K 117**	1					1		1	1	1							
**K 118**						1											
**K 120**						1							1				
**K 121**						1							1				
**K 122**						1		1					1				
**K 123**																	
**K 124**	1					1		1	1	1							
**K 126**						1		1	1				1				
**K 127**	1				1	1	1	1	1		1					1	1
**K 128**						1							1				
**K 129**						1		1	1		1						
**K 132**						1	1	1									
**K 133**						1							1				
**K 134**	1					1		1	1	1		1					
**K 135**						1											
**K 137**	1					1		1	1	1							
**K 138**						1		1									
**K 140**						1	1			1							
**K 141**	1					1		1	1	1							
**K 142**						1		1	1	1							
**K 160**						1							1				
**K 162**						1											
**U 001**																	
**U 002**						1											
**U 003**	1																
**U 004**						1											
**U 005**																	
**U 006**		1	1	1	1	1		1		1							
**U 007**						1			1								
**U 008**						1		1									
**U 009**						1	1			1							
**U 010**	1					1											
**U 011**						1		1									
**U 012**	1					1		1	1								
**U 013**	1	1	1	1	1	1		1	1	1	1						
**U 014**		1	1		1	1		1		1							
**U 015**						1		1	1								
**U 016**	1	1		1		1		1	1	1	1						
**U 017**						1											
**U 018**		1	1	1	1	1		1	1	1							
**U 019**																	
**U 020**							1		1	1							
**U 021**	1					1		1	1	1							
**U 022**								1	1								
**U 023**	1	1	1		1	1	1	1		1							
**U 024**						1											
**U 025**						1		1	1	1		1					
**U 026**	1	1	1	1	1	1		1	1	1	1						
**U 027**	1	1	1	1		1		1	1	1							
**U 028**	1					1		1									
**U 029**						1	1	1									
**U 030**		1	1		1	1		1		1							
**U 031**						1			1	1							
**U 032**																	
**U 033**	1	1	1	1	1	1		1		1							
**U 034**						1	1										
**U 035**	1	1	1	1	1	1		1		1							
**U 036**	1	1	1	1	1	1		1	1	1							
**U 037**						1	1	1									
**U 038**						1	1	1									
**U 039**	1	1				1		1	1	1							
**U 040**	1	1		1	1	1		1	1	1	1						
**U 041**						1											
**U 042**						1		1	1	1							
**U 043**									1	1		1					
**U 044**						1	1	1	1	1							
**U 045**						1											
**U 046**	1	1	1	1	1	1		1	1	1	1						
**U 047**		1	1	1	1	1		1		1							
**U 048**						1	1	1	1	1							
**U 049**		1	1	1	1	1		1	1	1							
**U 050**				1		1		1	1	1		1					
**U 051**						1			1	1							
**U 052**						1	1		1	1							
**U 053**						1	1		1	1							
**U 054**		1	1	1	1	1		1		1							
**U 055**						1		1									
**U 057**						1		1	1								
**U 058**		1	1	1	1	1		1		1							
**U 059**																	
**U 060**						1		1	1								
**U 061**						1			1	1							
**U 062**						1											
**U 063**	1		1	1	1	1		1									
**U 064**						1				1							
**U 066**	1	1	1		1	1		1		1							
**U 067**	1	1	1	1	1	1		1		1							
**U 068**						1											
**U 069**	1	1	1	1	1	1		1	1	1	1						
**U 070**						1											
**U 071**						1			1	1							
**U 072**	1	1	1	1	1	1		1	1	1	1						
**U 073**						1				1							
**U 074**						1											
**U 075**	1	1	1	1	1	1		1	1	1	1						
**U 076**						1				1							
**U 077**							1		1	1							
**U 079**	1	1	1	1		1		1		1							
**U 080**						1				1							
**U 081**						1		1	1							1	1
**U 082**		1	1	1	1	1		1		1							
**U 083**	1		1		1	1		1	1	1							
**U 084**	1	1	1	1	1	1		1	1	1	1						
**U 085**	1	1	1	1	1	1	1	1		1							
**U 086**		1		1		1		1	1	1						1	
**U 087**	1	1	1	1	1	1		1	1	1	1						
**U 088**		1	1	1	1	1		1									
**U 089**		1		1		1		1		1							
**U 090**	1	1	1	1	1	1	1	1		1							
**U 091**								1									
**U 092**																	
**U 093**	1					1		1	1	1							
**U 094**						1			1	1		1					
**U 095**	1					1		1	1	1							
**U 096**						1				1							
**U 097**	1	1				1		1	1	1							
**U 098**	1	1	1	1	1	1	1	1		1							
**U 099**						1		1		1							
**U 100**						1	1		1	1							
**U 101**	1	1	1	1	1	1		1	1	1	1						
**U 102**	1	1		1	1	1		1	1	1	1						
**U 103**	1					1			1		1						
**U 104**	1					1		1	1	1							
**U 105**	1	1	1	1	1	1		1		1							
**U 106**						1											
**U 107**					1	1											
**U 108**	1					1		1	1	1							
**U 109**						1											
**U 110**	1	1	1	1	1	1		1		1						1	
**U 111**	1	1	1	1	1	1		1	1	1	1						
**U 112**	1	1	1	1	1	1		1		1							
**U 113**						1	1										
**U 114**	1	1	1	1	1	1		1	1	1							
**U 115**						1				1							
**U 116**						1	1	1	1	1		1					
**U 117**						1		1	1	1		1					
**U 118**						1			1	1							
**U 119**	1				1	1		1	1								
**U 120**						1			1	1							
**U 121**						1			1	1							
**U 124**						1		1	1		1						
**U 125**	1	1	1	1	1	1	1	1		1							
**U 126**							1	1		1							
**U 127**				1		1		1	1	1							
**U 128**						1			1	1							
**U 130**						1											
**U 131**	1					1		1	1	1		1					
**U 134**						1				1							
**U 135**						1				1							
**U 136**						1		1	1	1							

### TRS-PCR profiling and construction of dendrograms

**Fig 1:**
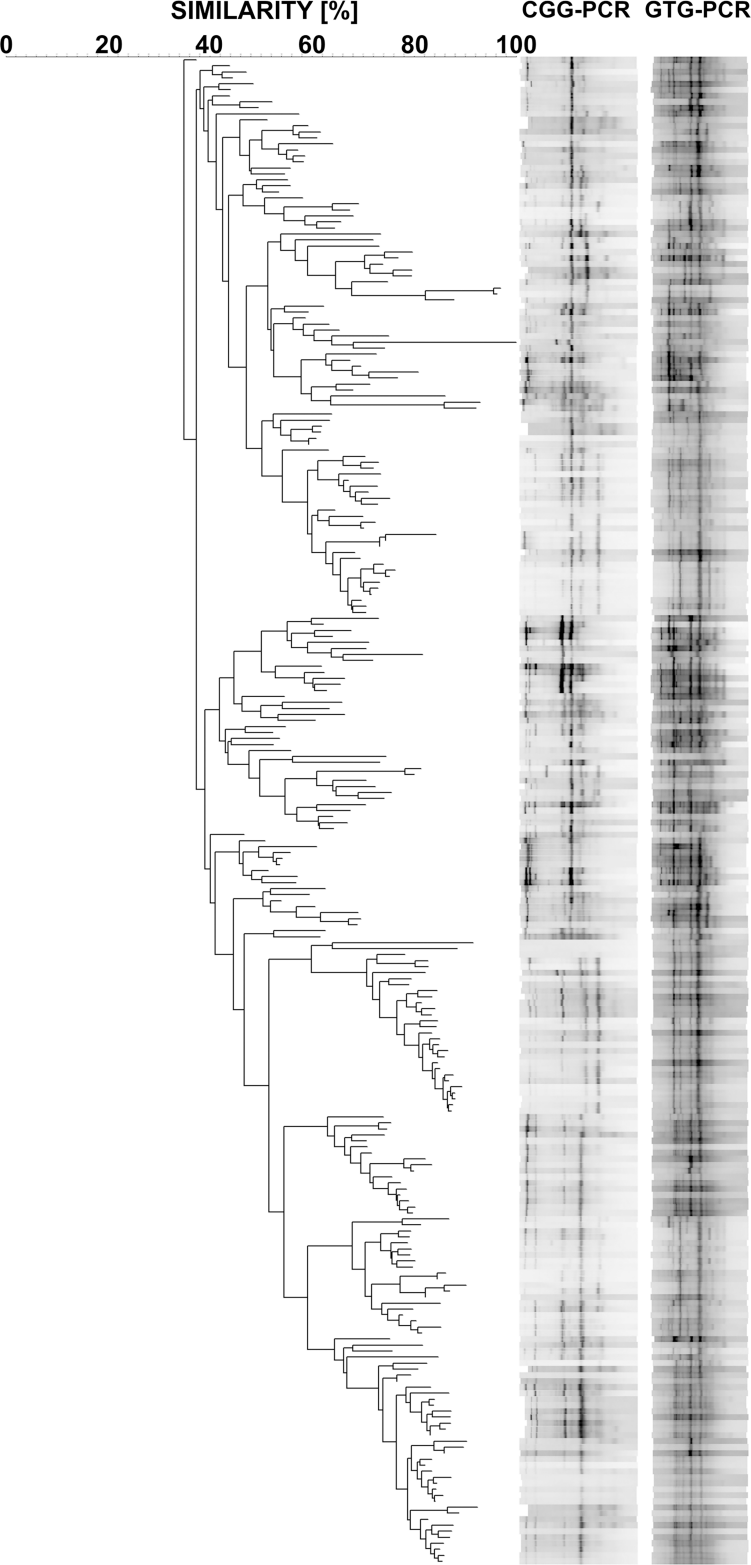
The average neighbor joining dendrogram constructed from the two trees based on the CGG-PCR and GTG-PCR profiling for 251 *E*. *coli* strains.

A collection of 251 genomic DNA samples were isolated from *E*. *coli* strains and TRS-PCR profiling using GTG and CGG primers was performed. Two TRS-PCR reactions were performed for each strain using primers containing GTG and CGG repeats respectively, according to procedures described elsewhere [9,10]. The PCR products, 8 μl of 50μl, were resolved by electrophoresis on 1.6% agarose gels (15×15 cm, 4 mm thick) in 1×Tris-acetate-EDTA (TAE) buffer, 2.5 V cm^-1^, until the dye (bromophenol blue) migrated 6 cm from the top of the gel. Such stringent conditions for the electrophoretic separations allow for carrying out trustworthy analyses. The DNA products for all of the primers ranged from 0.1 kbp to 2.5 kbp. The gels were stained with ethidium bromide (1 μg ml^-1^), visualized on a UV-transilluminator, and photographed (Fc8800, Alphainnotech). Subsequently, gels were optimized according to recommendations provided by BioNumerics version 5.00 software (Applied Maths, Belgium) and normalized with regard to a 100 bp Plus DNA size marker (Fermentas, Thermo Scientific Waltham, MA, USA). The CGG-PCR and GTG-PCR band profiles for each strain from the collection were obtained and respective dendrograms were constructed using the BioNumerics software (Pearson correlation, optimization 1%, position tolerance 1%). Finally, the average similarity Neighbor Joining dendrogram based on the two trees was assembled. The results are shown in Fig 1. Such dendrogram and virulence information were subsequently analyzed by our wrapper, around make.bisse() and find.mle() functions, R script.

### The BiSSE model

**Fig 2:**
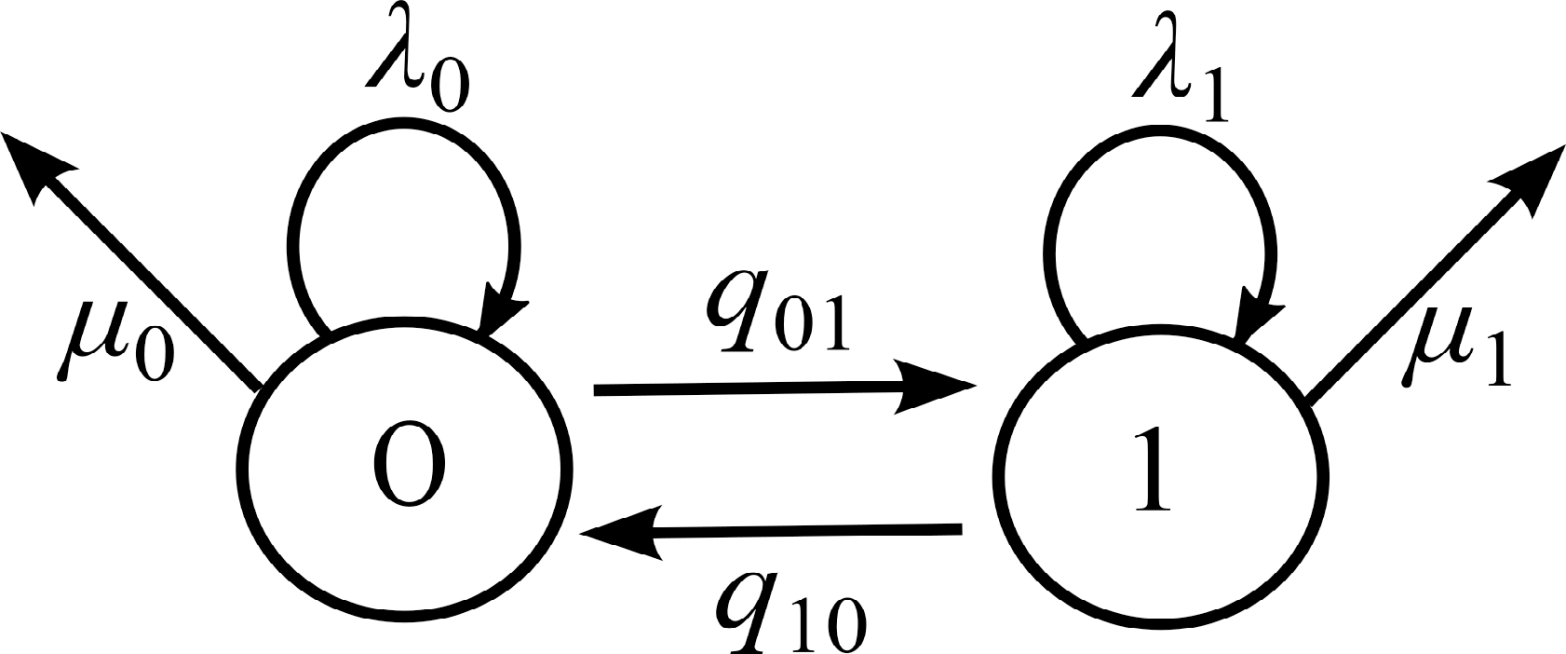
Graphical representation of the BiSSE model [11]. On each arrows the particular parameters of the BiSSE model were placed: *q*_01_, *q*10 –the transition between states; *λ*_0_, *λ*_1_ –the speciation rates; *μ*_0_, *μ*_1_ –the extinction rates. The state diagram has two states labeled 0 (non-pathogenic) and 1 (pathogenic).

In this study we used the BiSSE model [11] for binary states with four rate parameters. BiSSE models (Fig 2) the evolution of a binary trait (two possible states 0 and 1) and allows for estimation of the speciation (*λ*_0_, *λ*_1_), extinction (we assumed *μ*_0_ = *μ*_1_ = 0), and transition between states (*q*_01_, *q*_10_) rates. Knowledge of these rates sheds light on whether the trait controls diversification rates or not. In our case the trait levels correspond to non-pathogenic (0) and pathogenic (1). The transition rate from 0 to 1 is *q*_01_ and from 1 to 0 is *q*_10_. If the species is in state 0, then it has speciation rate *λ*_0_ and in state 1 speciation rate *λ*_1_.

Notice that in our setting we do not assume any extinction events, i.e. the extinction rates are set to 0, while in the general BiSSE model they can be non-zero. We may concisely describe the model as follows. Let *N*_0_(*t*) be the number of 0 strains at time *t* and *N*_1_(*t*) the number of 1 strains. Of course *N*(*t*) = *N*_0_(*t*)+*N*_1_(*t*) is the total number of strains present in the system at time *t*. We assume that at time 0, at the root of the tree there is one strain alive, *N*(0) = 1. We will estimate the root state, i.e. whether our data supports *N*_0_(0) = 1 or *N*_1_(0) = 1.

Immediately with the introduction of the BiSSE model there was concern about its power, i.e. its ability to distinguish between competing hypotheses of symmetric versus asymmetric models (given pairs of parameters equal versus not equal) [23]. Simulation studies indicated that a minimal sample size should be about 300 [23]. However, these investigations were done under the full six parameter BiSSE model. Later investigations (e.g. [24,25]) indicate that some questions can be analyzed based on much smaller samples. If some parameters are set to 0 then the power can increase dramatically and give sensible results with 100 species [24]. Asymmetric speciation rates can be detected with as few as 45 contemporary tip species [25,26]. In our setting the extinction rates are fixed at 0. Since these parameters are the most difficult to estimate [24,25], the consideration of a restricted sub-model should improve the situation. Quoting [24] p. 391, "… there are also many reasons for guarded optimism." especially as, quoting [25] "… low power should tend to reduce our ability to detect differences between parameters, rather than exacerbate them".

### The computer software and calculations

We wrote a wrapper script around the make.bisse() and find.mle() functions of the diversitree R package [13,14] that does model selection and then calculates the limit behavior of the model. We demonstrate the application of the BiSSE model to estimate parameters from genetic traits (see scheme of research hypothesis) and to illustrate this approach we estimate parameters from a collection of clinical *E*. *coli* strains. We used the diversitree R package to estimate four parameters (*λ*_0_, *λ*_1_, *q*_01_, *q*_10_) from the dendrograms. We considered various models: (*λ*_0_, *λ*_1_, *q*_01_, *q*_10_), (*λ*_0_, *λ*_1_, *q*_01_ = *q*_10_) and (*λ*_0_ = *λ*_1_, *q*_01_ = *q*_10_). This particular functionality is actually available through the diversitree::constrain() function. However, our wrapper function is more general and allows the user to specify an arbitrary parametrization of BiSSE's parameters. In particular we do not have the restrictions "Terms that appear on the right hand side of an expression may not be constrained in another expression, and no term may be constrained twice." (from diversitree::constrain()’s help). Our wrapper function should be useful to researchers as it seems that biological studies can require restricted BiSSE setups (e.g. [24,25]).

Model selection was done using AICc [27]. Assessment of model fit was done by comparing the observed fractions of pathogenic strains to the composite parameter *P*_1_ (see Section Probability of maintaining the VF in *E*. *coli* strains) in Table 3. Furthermore, in Table 3 we can see that the Taylor expansion approximation (see Section Probability of maintaining the VF in *E*. *coli* strains) of *P*_1_ corresponds well to the theoretical and observed proportions. The estimation of the four parameters was based on the provided phylogeny and observed states. From the estimated parameters we extracted, using an R script, the almost sure limits of the proportion of the VF in *E*. *coli* strains (see S1 Appendix).

**Table 3:** The comparison of parameter *P*_1_ to the different proportions: *N*_1_/(*N*_1_+*N*_0_); *q*_01_/*λ*_0_; *λ*_0_/*λ*_1_ for particular virulence factors (VF gene). The *P*_1_ denotes probability of maintaining the virulence factors in *E*. *coli* strains. K and U define the isolation environment, stool and urine, respectively.

VF gene	*P*_1_	*N*_1_/(*N*_1_+*N*_0_)	*q*_01_/*λ*_0_	*λ*_0_/*λ*_1_
*astA*-K	0,5	0,056911	0,062749222	1
*astA*-U	0,162009059	0,15625	0,166929019	5,923011389
*cnf1*-K	0,026247741	0,02439	0,026974121	36757,41693
*cnf1*-U	0,562593524	0,296875	0,07770742	0,474817981
*fimG*-K	0,5	0,97561	0,017012576	1
*fimG*-U	0,892325172	0,898438	0,494043	0,198624374
*fyuA*-K	0,5	0,601626	0,328658853	1
*fyuA*-U	0,681386744	0,609375	0,86451878	0,409062184
*hly1*-K	0,059809609	0,04065	0,063872785	1196660,446
*hly1*-U	0,635089603	0,3125	0,091917429	0,466567334
*iroN*-K	0,281900561	0,292683	0,287407517	0,441978106
*iroN*-U	0,704457027	0,65625	2,458813734	0,171547728
*iutA*-K	0,691463682	0,626016	6696,322014	8,32E-05
*iutA*-U	0,516360148	0,484375	1,185543854	0,290593584
*papC*-K	0,278749076	0,268293	0,459292231	123,6434019
*papC*-U	0,300632736	0,351563	0,120384425	0,275223691
*sat*-K	0,783173018	0,373984	0,154136119	0,660475068
*sat*-U	0,111249184	0,117188	0,126134155	0,235297811
*sfa*-K	0,016700084	0,01626	0,016986588	8386,222371
*sfa*-U	0,694965581	0,335938	0,073105204	0,498620108
*tsh*-K	0,101117086	0,065041	0,113933518	591123,6118
*tsh*-U	0,064591836	0,054688	0,069382734	532869,461
*usp*-K	0,016700084	0,01626	0,016986588	8386,222371
*usp*-U	0,623225843	0,3125	0,080864901	0,481605114

All calculations were done in R on the multicore computational server of the Department of Mathematics Uppsala University (R 3.2.5 for Ubuntu 12.04.5 LTS on a 1.4GHz. AMD Opteron Proc. 6274). We ran the computation on 4 cores and the whole analysis took about 3 days.

Source code and sample data freely available for download at https://github.com/BISSE-TRS/ppbEcoli, distributed under the GNU GPLv3 license.

## Results

### Research hypothesis

**Fig 3:**
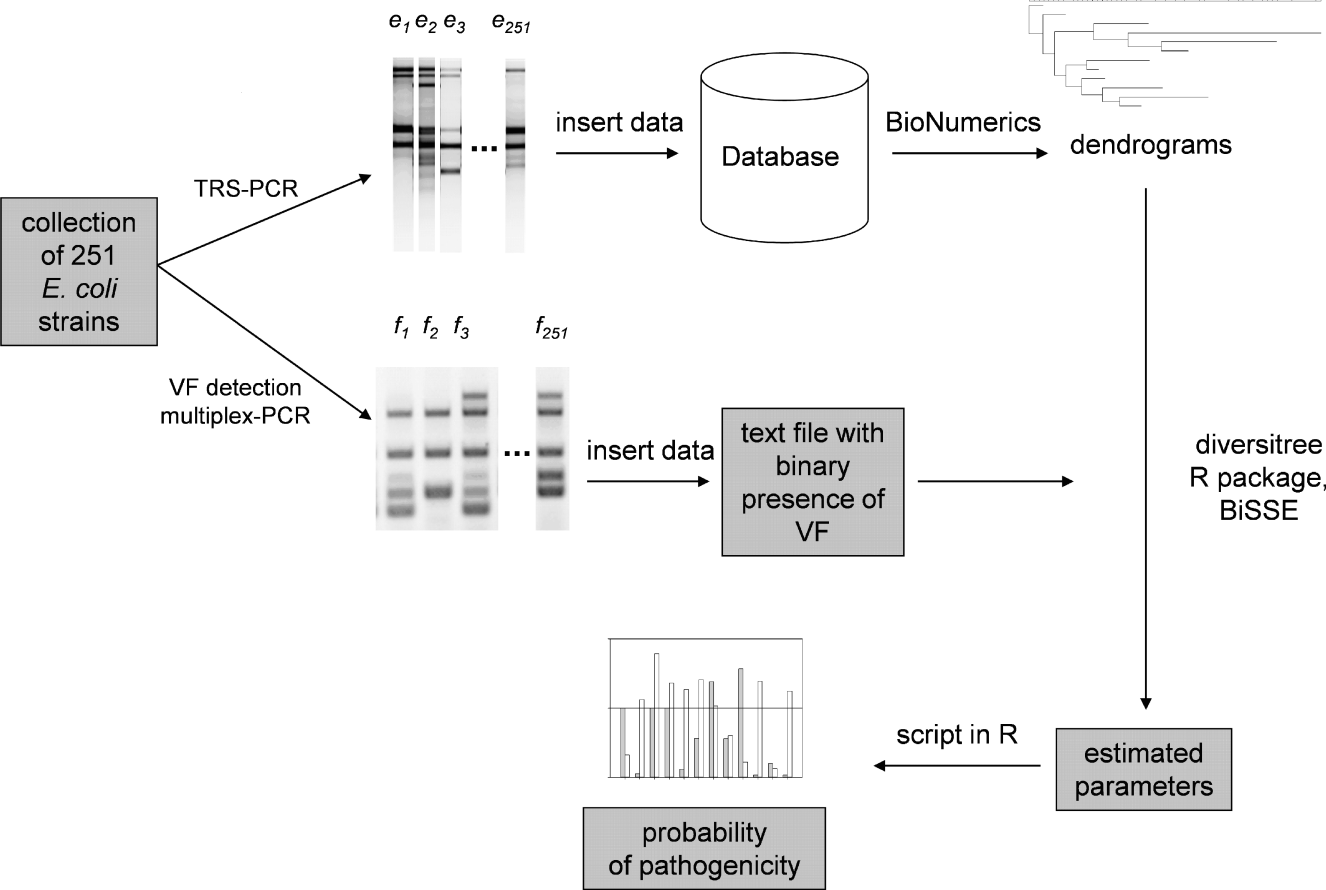
The concept of the investigation. Each strain of *E*. *coli* has been assigned an individual profile of the TRS-PCR and a set of virulence traits (see Materials and Methods). The method of predicting pathogenicity relies on using BiSSE model and microsatellites TRS-PCR profiling. Additionally the wrapper scripts calculate probability of pathogenicity.

In this work, we ask whether, with the disposal of dendrograms based on TRS profiles and the BiSSE method, it is possible to predict the maintenance of particular VF features in a population. A diagram summarizing our work is presented in research hypothesis, Fig 3.

### The estimated rates of speciation rates - *λ*_0_, *λ*_1_

**Fig 4:**
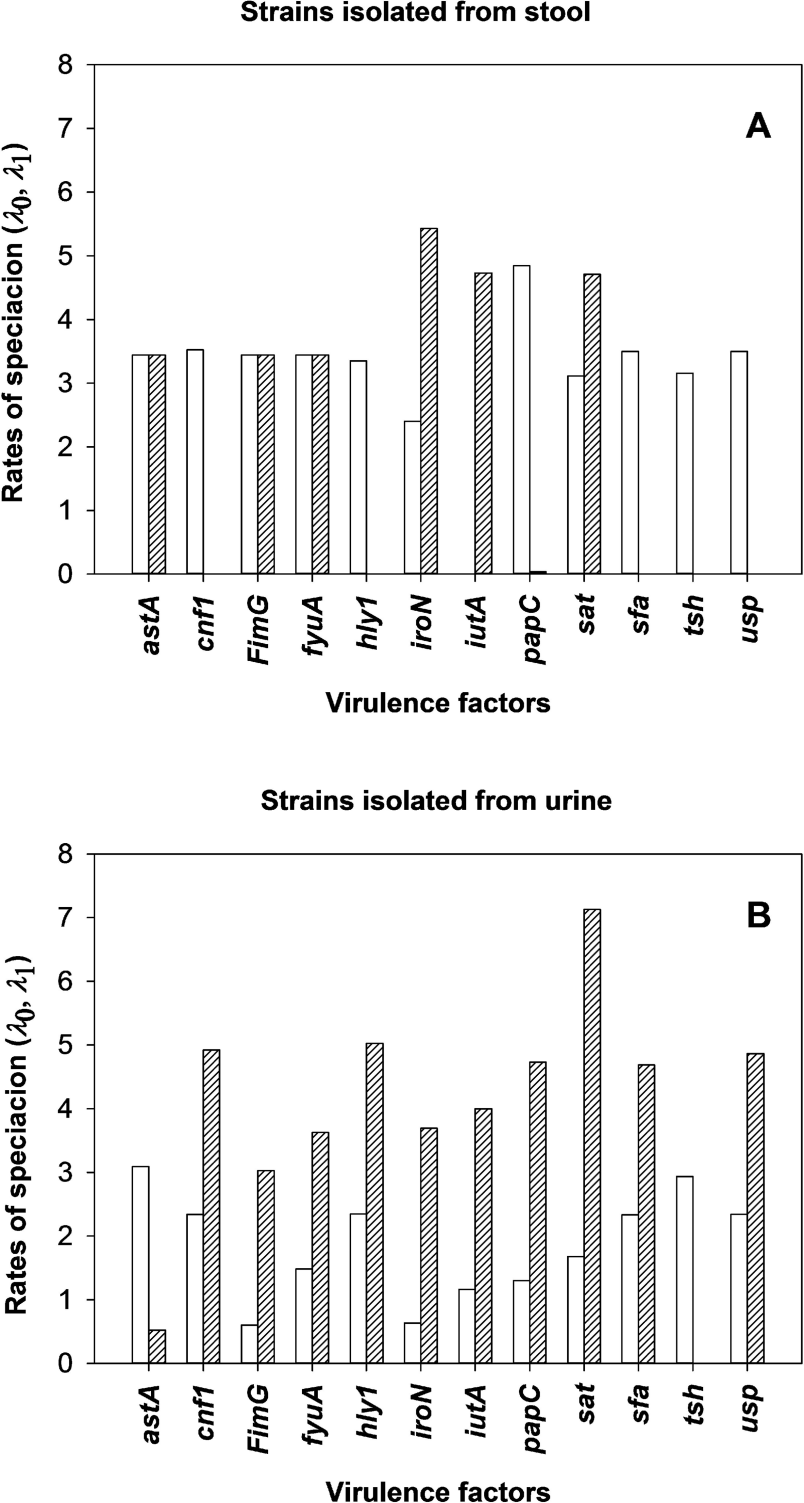
The rates of speciation depending on the absence or possessing the given trait of virulence for collection of strains isolated from stool (A) and urine (B). Open bars–lack of the VF (*λ*_0_); hatched bars–presence of the VF (*λ*_1_).

In our study we take the viewpoint that strains are genetically variable but do not go extinct in a population. Extinction is a principle of evolution, but this phenomenon is attributed to species. In our case we do not have classical extinction of species present. Rather, we observe that with time the bacterial genetic pool of strains becomes more diverse. Hence, we focus on the no extinction model (μ_0_ = μ_1_ = 0) [28,29]. Even though BiSSE is known to have low power for samples less than 300, Maliska et al. [25] indicated the asymmetries in speciation rates can be detected with as few as 45 species. Hence, their estimates are a primary focus of our understanding of VF dynamics in *E*. *coli*. Here, we demonstrated differences in rates of speciation depending on the absence (*λ*_0_) or presence (*λ*_1_) of the given trait of virulence. Fig 4A shows results obtained for intestinal *E*. *coli* strains and Fig 4B shows results for strains isolated from urine. In the case of strains isolated from stool samples a higher rate of propagation can be observed for those not possessing *cnf1*, *hly1*, *papC*, *sfa*, *tsh and usp* genes. It is not surprising given that such virulence factors (except *tsh*) typically occur in uropathogens [1,30–32]. On the other hand, possession of *iron* and *iutA* resulted in much higher rate of propagation. In the case of strains isolated from urine most of the virulence factors had a stimulating effect on dissemination of strains except for *astA* and *tsh* genes. One could expect this, as urine is not naturally inhabited by microorganisms and therefore, numerous virulence factors facilitate colonization.

### The transition between states rates–*q*_01_, *q*_10._

**Fig 5:**
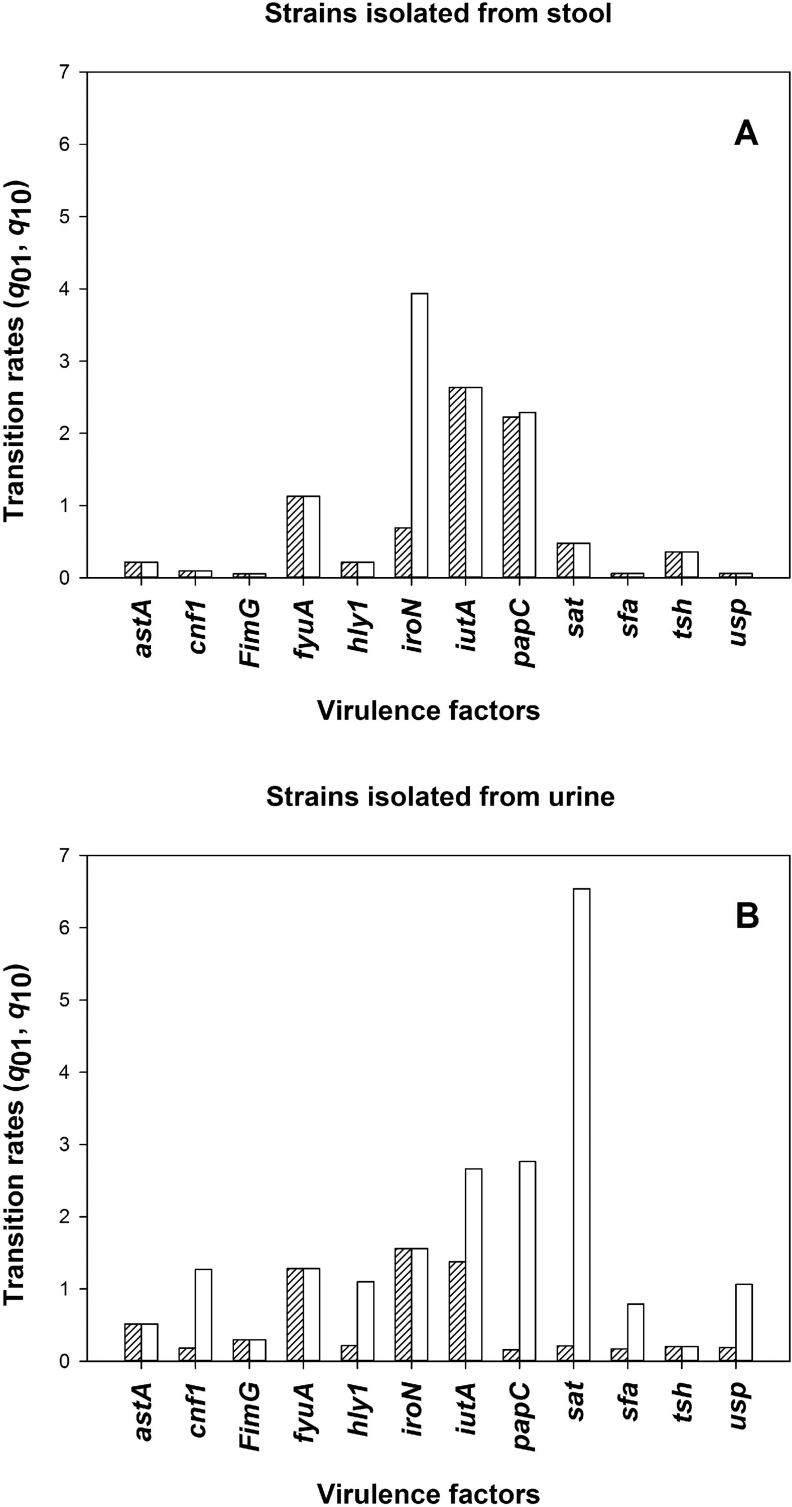
The transition rates between states–*q*_01_, *q*_10_ for collection of strains isolated from stool (A) and urine (B). Open bars–non-pathogenic direction (*q*_10_); hatched bars–pathogenic direction (*q*_01_).

Here we studied rates of mutation in pathogenic (*q*_01_) and non-pathogenic (*q*_10_) directions for strains isolated from stool (Fig 5A) and urine (Fig 5B). Interestingly, in both cases when differences were pronounced the *q*_10_ transition was preferred. This is consistent with the fact that maintenance of a VF is energetically costly for microorganisms and additionally, lack of the virulence factor allows for “hiding” from the host’s immunological defense system. Furthermore, highly virulent strains may sensitize individuals allowing for recurrent infections caused by these less virulent strains [1,33].

### Probability of maintaining the VF in *E*. *coli* strains

**Fig 6:**
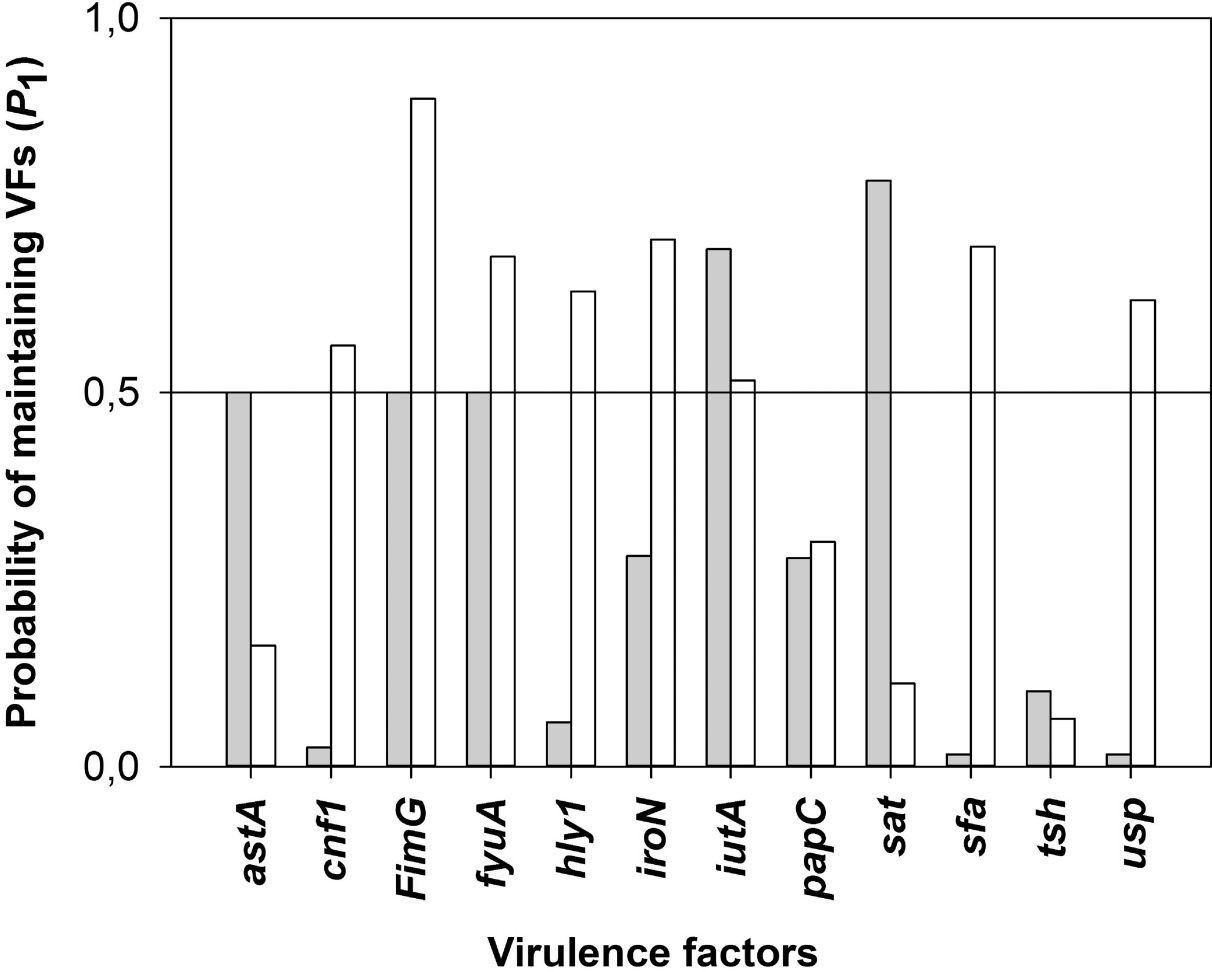
The Probability (*P*_1_) of maintaining the virulence factors in *E*. *coli* strains isolated from stool (hatched bars) and urine samples (open bars). Values above 0,5 (horizontal black line) indicate higher than 50% chance for being maintained.

Based on the estimated BiSSE rates it was possible to estimate the long term proportions (*P*_1_: = *v*_1_/(*v*_0_+*v*_1_), see S1 Appendix, Thm. 2.2) of the VF features in the populations. The results are shown in Fig 6. Among analyzed VF features the following traits had a higher than 50% chance for being maintained in an *E*. *coli* population–*cnf1*, *fimG*, *fyuA*, *hly1*, *iroN*, *iutA*, *sat*, *sfa* and *usp*. The vast majority of these traits exhibited pathogenicity maintenance in the strains isolated from urine. This seems to be justified by the fact that the VFs mentioned above are necessary for the colonization of the urinary tract in humans i.e. adhesins (*fimG*, *sfa*), toxins (*hly1*, *cnf1*, *sat*), iron uptake system (*fyuA*, *iutA*, *iroN*) and bacteriocin (*usp*) [1,31,32,34]. These VFs, however, are not necessary for the development of intestinal pathogens. If the non-pathogenic strains speciate faster than the pathogenic ones (i.e. *λ*_0_>*λ*_1_), then a Taylor expansion of *P*_1_ points to a very simple formula for it: *q*_01_/*λ*_0_ (provided this is lesser than 1). In Table 3 we also included this simplified calculation.

## Discussion

In this paper we presented a comprehensive approach for predicting pathogenicity in a population based on a state dependent model and TRS-PCR profiling. Additionally, this paper shows that it is possible to apply this approach to real laboratory genetic data–from 251 *E*. *coli* strains. Our first research goal was to infer dendrograms for the *E*. *coli* population. This required the gathering of a unique collection of bacterial populations (251 strains) and a detailed laboratory genetic analysis, including CGG- and GTG-PCR profiling as well as the identification of pathogenic traits. Next, we applied the BiSSE model to such a collection of genetic data. Any BiSSE analysis of biological data runs the risk of low power and one should be careful with drawing conclusions. However, in our case there is place for "guarded optimism” as we restrict our model by excluding the two parameters most difficult to estimate (the extinction rates). We used AICc to distinguish between competing models and remembering that "… *low power should tend to reduce our ability to detect differences between parameters*, *rather than exacerbate them*" [25] we notice that most VFs have equal transition rates. The exceptions to this are *hly1* (in U), *iroN* (in K) *iutA* (in U), *papC* (both U and K), *sat* (in U), *sfa* (in U), *usp* (in U), *cnf1* (in U). In all of these cases we have *q*_10_>*q*_01_, i.e. the loss of pathogenicity is favoured. Furthermore we can see that asymmetry (loss of pathogenicity) is preferred in the urine environment. Such a behavior was previously observed by others [1,33].

Our computational results confirmed previous biological observations demonstrating a prevalence of some virulence traits in specific bacterial sub-groups [21,30]. The necessity of harboring some VFs in *E*. *coli* pathogens was indicated. For example, UPEC strains exist within the intestinal tract of humans but possess specific factors (adhesins, toxins, siderophores and bacteriocins) that permit their successful transition from the intestines to the urine tract. These VFs are encoded by genes located at the selected regions of chromosomal DNA, plasmids and/or transposons, named pathogenicity islands (PAIs). PAIs are flexible genetic elements, holding the mobility sequences, which are transferred horizontally between the bacterial cells [2,3]. This phenomenon is significant for bacterial population evolution/diversity. Additionally, it allows for VFs’ synergy during the process of pathogenicity. For example, *iroN* and *sfa* are located on PAI III in *E*. *coli* 536 and *hly* and *cnf1* are encoded by PAI II in *E*. *coli* J96. It may suggest that these VF pairs will be co-transmitted. However, in our study only 35,8% of strains harboring *iroN* encodes also *sfa* and 86,6% of strains encoded for both *hly* and *cnf1*. In the latter case however, we need to keep in mind that alpha-haemolysin gene cluster is present also on plasmids and the other PAIs, some of which do not encode the CNF-1 [35]. In addition, one needs to remember that not always the PAIs are transmitted completely and due to recombination errors, some sets of features may not be lost or acquired jointly [28].

As mentioned above, our research has been conducted using 251 *E*. *coli* strains that included two collections–from urine and stool samples. These collections were not equal in terms of their virulence factors repertoires (Table 1) therefore, it would be interesting to extend this research to more strains harboring numerous intestinal VFs. Since the population studied was divided into two collections isolated from two different environments one could also consider the GeoSSE model to capture potential differences between the urine and stool environments. However, on the one hand the sample size of 251 is probably too small for such a complex model (10 parameters, even with extinction set to 0). On the other hand the BiSSE model is a submodel of GeoSSE. GeoSSE has separate BiSSE models in each environment and then transition rates between the environments. Hence, as we analyze the two environments separately ignoring the transitions, the use of GeoSSE would probably result in noticing finer details in the data, i.e. studying it with a tool that has a higher resolution-the interaction between the environments. BiSSE allows us to observe more general properties inside each environment. These are already consistent with biological intuition.

Additionally, this paper presents a method of estimating the probability of persistence of the VF in *E*. *coli* strains. Noteworthy, this is a comprehensive approach and it may be used to predict pathogenicity of other bacterial taxa. We believe that our developed software should be useful for biologists that want to use restricted BiSSE models or who want to parametrize the parameters. Our wrapper function seems to be flexible enough for such purposes.

### Conclusions

The binary state dependent model and TRS-profiling appear to be useful tools for predicting persistence of pathogenicity in an *E*. *coli* population.

## Supporting information

S1 Appendix Predicting pathogenicity behavior in E. coli population through a state-dependent model and TRS profiling. (PDF)
