# Supplementary material for: Predicting pathogenicity behavior in Escherichia coli population through a state dependent model and TRS profiling
Source: PLoS Comput Biol. 2018 Jan 31;14(1):e1005931. doi: 10.1371/journal.pcbi.1005931 (PMC5809097; doi:10.1371/journal.pcbi.1005931)
Supplement: S1 Appendix — (PDF) [file pcbi.1005931.s001.pdf]

S1 Appendix to Predicting pathogenicity behavior in *E. coli* population through a state–dependent model and TRS profiling

Krzysztof Bartoszek, Marta Majchrzak, Sebastian Sakowski,
Anna B. Kubiak–Szeligowska, Ingemar Kaj, Paweł Parniewski

July 10, 2017

1 The BiSSE model

We consider a model for particles of two types – type 0 and type 1. At time t there are $N(t) = N_0(t) + N_1(t)$ particles, where $N_i(t)$, $i = 0, 1$, is the number of type i particles. Each particle can split (with constant rate λ_i), die (with constant rate μ_i) or change type (with constant rate q_{i1-i}) independently of all the other particles. Initially there is a single particle of type either 0 or 1. This is a discrete–state–continuous–time–Markov–chain, which is concisely described by its transitions

$$(N_0(t+\Delta t), N_1(t+\Delta t)) = \begin{cases} (N_0(t)+1, N_1(t)-1) & \text{with probability} & q_{10}N_1(t)\Delta t + o(\Delta t) \\ (N_0(t)-1, N_1(t)+1) & -\text{“}- & q_{01}N_0(t)\Delta t + o(\Delta t) \\ (N_0(t)+1, N_1(t)) & -\text{“}- & \lambda_0 N_0(t)\Delta t + o(\Delta t) \\ (N_0(t), N_1(t)+1) & -\text{“}- & \lambda_1 N_1(t)\Delta t + o(\Delta t) \\ \text{other change} & -\text{“}- & o(\Delta t) \end{cases} \tag{1}$$

Let $p_{n_0,n_1}(t)$ be the probability that $N_0(t) = n_0$ and $N_1(t) = n_1$. From Eq. (1) we obtain the following infinite system of ODEs:

$$\begin{aligned} p'_{n_0,n_1}(t) &= -\left((q_{01}+\lambda_0)\, n_0 + (q_{10}+\lambda_1)\, n_1\right) p_{n_0,n_1}(t) \\ &\quad + q_{10}(n_1+1)p_{n_0-1,n_1+1}(t) + q_{01}(n_0+1)p_{n_0+1,n_1-1}(t) \\ &\quad + \lambda_0(n_0-1)p_{n_0-1,n_1}(t) + \lambda_1(n_1-1)p_{n_0,n_1-1}(t). \end{aligned} \tag{2}$$

Using Eq. (2) we can write down [see e.g. p.69 Bailey, 1964] the probability generating function for (N_0, N_1),

$$G(z_0, z_1, t) = \mathrm{E}\left[z_0^{N_0(t)} z_1^{N_1(t)}\right] = \sum_{n_0,n_1} p_{n_0,n_1}(t) z_0^{n_0} z_1^{n_1}. \tag{3}$$

The probability generating function of our system is the solution to the PDE,

$$\frac{\partial G}{\partial t} = -\left((q_{01}(z_0 - z_1) + \lambda_0 z_0(1-z_0)) \frac{\partial G}{\partial z_0} + (q_{10}(z_1 - z_0) + \lambda_1 z_1(1-z_1)) \frac{\partial G}{\partial z_1} \right) \tag{4}$$

or in vector form,

$$\frac{\partial G}{\partial t} = -\left(q_{01}(z_0 - z_1) + \lambda_0 z_0(1-z_0), q_{10}(z_1 - z_0) + \lambda_1 z_1(1-z_1)\right) \cdot \mathrm{D}_z G,$$

with initial conditions,

$$\begin{array}{rcl} G(z_0, z_1, 0) & = & p_{10}(0) z_0 + p_{01}(0) z_1, \\ \frac{\partial}{\partial z_0} G(0, z_1, t) & = & p_{1,0}(t), \\ \frac{\partial}{\partial z_1} G(z_0, 0, t) & = & p_{0,1}(t). \end{array} \quad (5)$$

We recognize this as a transport equation and using the technique presented in §2.1 by Evans [1998] we write

$$z(s) = G(h(s, z_0, z_1), t+s), \quad s \in \mathrm{R}$$

where the function $h(s, z_0, z_1) : \mathbb{R}^3 \to \mathbb{R}^2$ is such that

$$z'(s) = D_z G(h(s, z_0, z_1), t+s) \cdot h'(s, z_0, z_1) + G_t'(h(s, z_0, z_1), t+s) = 0.$$

This entails that,

$$\begin{array}{rcl} \frac{\mathrm{d}h_0(s,z_0,z_1)}{\mathrm{d}s} & = & -(-\lambda_0 h_0(s, z_0, z_1)^2 + (\lambda_0 + q_{01}) h_0(s, z_0, z_1) - q_{01} h_1(s, z_0, z_1)) \\ \frac{\mathrm{d}h_1(s,z_0,z_1)}{\mathrm{d}s} & = & -(-\lambda_1 h_1(s, z_0, z_1)^2 + (\lambda_1 + q_{10}) h_1(s, z_0, z_1) - q_{10} h_0(s, z_0, z_1)) \end{array}$$

with initial condition $h(0, z_0, z_1) = (z_0, z_1)$. Assuming we could solve the above system and find h, then the probability generating function G will be

$$G(z_0, z_1, t) = p_{10}(0) h_0(-t, z_0, z_1) + p_{01}(0) h_1(-t, z_0, z_1). \quad (6)$$

Since the quadratic system of ODEs for h does not seem to have a closed form solution, the likelihood of the system is intractable. Therefore, estimation software for the BiSSE–type models [FitzJohn, 2010, 2012, Maddison et al., 2007, FitzJohn et al., 2009] evaluates the likelihood through a numerical solution of an appropriate ODE system.

However, we can use Eq. (2) to find equations for all the moments of the system. In particular, the first and second order moments satisfy the linear system of ODEs

$$\begin{bmatrix} \mathrm{E}[N_0(t)]' \\ \mathrm{E}[N_1(t)]' \\ \mathrm{E}[N_0^2(t)]' \\ \mathrm{E}[N_1^2(t)]' \\ \mathrm{E}[N_0(t)N_1(t)]' \end{bmatrix} = \begin{bmatrix} \lambda_0 - q_{10} & q_{10} & 0 & 0 & 0 \\ q_{01} & \lambda_1 - q_{10} & 0 & 0 & 0 \\ \lambda_0 + q_{01} & q_{10} & 2(\lambda_0 - q_{01}) & 0 & 2q_{10} \\ q_{01} & \lambda_1 + q_{10} & 0 & 2(\lambda_1 - q_{10}) & 2q_{01} \\ -q_{01} & -q_{10} & q_{01} & q_{10} & \lambda_0 + \lambda_1 - q_{01} - q_{10} \end{bmatrix} \begin{bmatrix} \mathrm{E}[N_0(t)] \\ \mathrm{E}[N_1(t)] \\ \mathrm{E}[N_0^2(t)] \\ \mathrm{E}[N_1^2(t)] \\ \mathrm{E}[N_0(t)N_1(t)] \end{bmatrix}. \quad (7)$$

2 Almost sure limit

Even though the likelihood is not tractable in closed form, Janson [2004] characterized the almost sure behaviour and provided central limit theorems for such multitype branching processes of which the BiSSE model is a particular case. Further notable results for similar models are due to Jagers [1969], Sagitov and Serra [2009], Yakovlev and Yanev [2009, 2010], Antal and Krapivsky [2010].

It is worth pointing out that when one of the transition rates (either q_{01} or q_{10}) is 0, then Antal and Krapivsky [2010] found a closed form expression for the likelihood. We now describe [following Janson, 2004] the almost sure behaviour of the BiSSE process. In particular it tells us how the process will stabilize, and what proportion of type 0 and type 1 particles are expected after long time.

We first introduce two key matrices related to the BiSSE model. These two matrices summarize the average behaviour of the model. The first is the mean offspring matrix,

$$\mathbf{M}_o = \begin{bmatrix} \lambda_0 - q_{01} & q_{01} \\ q_{10} & \lambda_1 - q_{10} \end{bmatrix}$$

that comes from the ODE system of Eq. (7).

The second more important for us is derived from the mean change matrix [as considered by Janson, 2004]

$$\mathbf{M}_c = \begin{bmatrix} \frac{\lambda_0 - q_{01}}{\lambda_0 + q_{01}} & \frac{q_{01}}{\lambda_0 + q_{01}} \\ \frac{q_{10}}{\lambda_1 + q_{10}} & \frac{\lambda_1 - q_{10}}{\lambda_1 + q_{10}} \end{bmatrix}.$$

$\mathbf{M}_c$ stems from how the system can change, if type 0 is drawn, then the change is one of $\{(1,0),(-1,1)\}$ and if type 1 then $\{(1,-1),(0,1)\}$, with the conditional probabilities clearly seen in $\mathbf{M}_c$. Entry i,j of $\mathbf{M}_c$ tells us by how much (in expectation) will the population of j–types change if an individual of type i split. We further introduce an activity vector

$$a = \begin{pmatrix} \lambda_0 + q_{01}, & \lambda_1 + q_{10} \end{pmatrix}$$

whose entries give us the respective intensities of lifetimes of types 0 and 1, or alternatively, given that an event occurred, the probability that it concerned type i is $a_i N_i(t)/(a_0 N_0(t) + a_1 N_1(t))$. Following [Janson, 2004] we introduce the matrix

$$\mathbf{A} := [\mathbf{A}(i,j)]_{i,j=1}^{2} = [a(j)\mathbf{M}_c(j,i)]_{i,j=1}^{2}$$

which equals

$$\mathbf{A} = \begin{bmatrix} \lambda_0 - q_{01} & q_{10} \\ q_{01} & \lambda_1 - q_{10} \end{bmatrix}.$$

The eigenvalues of $\mathbf{A}$ are

$$\begin{aligned} \gamma_- &= \tfrac{1}{2}\left(\lambda_0 + \lambda_1 - q_{01} - q_{10} - \sqrt{(\lambda_0 - \lambda_1 - q_{01} + q_{10})^2 + 4q_{01}q_{10}}\right), \\ \gamma_+ &= \tfrac{1}{2}\left(\lambda_0 + \lambda_1 - q_{01} - q_{10} + \sqrt{(\lambda_0 - \lambda_1 - q_{01} + q_{10})^2 + 4q_{01}q_{10}}\right) \geq 0, \end{aligned}$$

and the right eigenvectors

$$\begin{aligned} \tilde{v}_- &= \left(-\tfrac{1}{2q_{01}}\left(\lambda_1 - \lambda_0 + q_{01} - q_{10} + \sqrt{(\lambda_0 - \lambda_1 - q_{01} + q_{10})^2 + 4q_{01}q_{10}}\right), 1\right), \\ \tilde{v}_+ &= \left(-\tfrac{1}{2q_{01}}\left(\lambda_1 - \lambda_0 + q_{01} - q_{10} - \sqrt{(\lambda_0 - \lambda_1 - q_{01} + q_{10})^2 + 4q_{01}q_{10}}\right), 1\right). \end{aligned}$$

Let us introduce the following notation

$$\begin{aligned} D &= \lambda_1 + \lambda_0 - q_{01} - q_{10}, \\ H &= \lambda_0 - \lambda_1 - q_{01} + q_{10}, \\ \Delta &= (\lambda_0 - \lambda_1 - q_{01} + q_{10})^2 + 4q_{01}q_{10} = H^2 + 4q_{01}q_{10} \geq 0, \\ S &= \lambda_0 + \lambda_1 + q_{01} + q_{10}. \end{aligned}$$

Now the eigenvalues can be written as,

$$\begin{aligned}\gamma_- &= \tfrac{1}{2}\left(D-\sqrt{\Delta}\right),\\ \gamma_+ &= \tfrac{1}{2}\left(D+\sqrt{\Delta}\right)\geq 0.\end{aligned}$$

The right eigenvectors (as returned by Mathematica 9.0, for Linux x86 (64–bit) running on Ubuntu 12.04.5 LTS, when $\Delta \neq 0$) are

$$\begin{aligned}\tilde{v}_- &= \left(\tfrac{1}{2q_{01}}\left(H-\sqrt{\Delta}\right),1\right),\\ \tilde{v}_+ &= \left(\tfrac{1}{2q_{01}}\left(H+\sqrt{\Delta}\right),1\right).\end{aligned}$$

The left eigenvectors (calculated as $\mathbf{P}^{-T}$, where $\mathbf{P}$ is the matrix whose columns are $\mathbf{A}$'s right eigenvectors and when $\Delta \neq 0$) are

$$\begin{aligned}\tilde{u}_- &= \left(\tfrac{-q_{01}}{\sqrt{\Delta}},\tfrac{\sqrt{\Delta}+H}{2\sqrt{\Delta}}\right),\\ \tilde{u}_+ &= \left(\tfrac{q_{01}}{\sqrt{\Delta}},\tfrac{\sqrt{\Delta}-H}{2\sqrt{\Delta}}\right).\end{aligned}$$

We can immediately see that γ_+ is the dominating eigenvalue and it has to be positive (0 only in the degenerate case of all model parameters equalling 0) which on the other hand is obvious as $\mathbf{M}_c$ is a strictly positive matrix. Athreya and Ney [2004], Janson [2004] consider normalized eigenvectors,

$$u_+ \cdot v_+ = 1 \qquad v_+ \cdot a = 1$$

[Athreya and Ney, 2004, had $a = (1,1)$]. Mathematica already returns eigenvectors such that $\tilde{v}_+ \cdot \tilde{u}_+ = 1$ so it remains to normalize $\tilde{v}_+$. We have

$$\tilde{v}_+ \cdot a = \frac{\lambda_0 + q_{01}}{2q_{01}}\left(H+\sqrt{\Delta}\right) + \lambda_1 + q_{10} = B$$

and now define

$$v_+ = \frac{1}{B}\tilde{v}_+ \qquad u_+ = B\tilde{u}_+.$$

Athreya and Ney [2004] provide us with the following results.

Theorem 2.1. *[Thm 2 Ch 7.5 Athreya and Ney, 2004]*

$$\lim_{t\to\infty}(N_0(t),N_1(t))e^{-\gamma_+ t} = Wv_+$$

where W is a random variable with expectation equalling $v_+(i)$, $i = 0,1$ depending on the initial type.

We now explicitly list the assumptions that Janson [2004] makes about the branching multitype branching process.

(A1) If a branching event occurs in a type i particle, the resulting number of type i particles may decrease by at most 1 while the number of particles of any other type can only increase.

(A2) The second moment of the change in type j particles, given that type i was affected, is finite.

(A3) The largest eigenvalue, γ_+, of $\mathbf{A}$ is positive.

(A4) The largest eigenvalue, γ_+, of $\mathbf{A}$ is simple.

(A5) There exists a dominating type i, i.e. a type such that $N_i(0) > 0$. *In our case it means that we will condition on the root being in a given state.*

(A6) The eigenvalue, γ_+, belongs to the dominating type. We say a type i is dominating if for every type j there is a possibility to find a type j eventually if we start with a single type i. *In our case this means that both transition rates are positive.*

Given assumptions (A1)–(A6) we translate Janson [2004]'s Theorem 3.15, concerning the limit frequencies, to the BiSSE model case. Notice that all of the six assumptions are met in the BiSSE model case. Let $(b_0, b_1) =: b \in \mathbb{R}^2$ and $z \geq 0$ and define the stopping time

$$\tau_b(z) := \min\{t \geq 0 : b \cdot (N_0(t), N_1(t)) \geq z\},$$

i.e. the first time $b_0 N_0(t) + b_1 N_1(t)$ exceeds z. In particular taking $z = n$ and $b = (1, 1)$ gives us the first time when there are n contemporary species. Obviously as $\gamma_+ > 0$ and we assumed no extinction we have $N_0(t) + N_1(t) \to \infty$ almost surely.

Theorem 2.2. *[cf. Theorem 3.15 Janson, 2004] Under assumptions (A1)–(A6)*

$$n^{-1}(N_0(\tau_{(1,1)}(n)), N_1(\tau_{(1,1)}(n))) \xrightarrow{1} (v_0, v_1)/(v_0 + v_1) = \left(\frac{H + \sqrt{\Delta}}{H + \sqrt{\Delta} + 2q_{01}}, \frac{2q_{01}}{H + \sqrt{\Delta} + 2q_{01}}\right).$$

This limit is the same as in the unconditioned case [Ch. 1, Thm 8.3 Mode, 1971]. By uncoditioned we mean that time goes to infinity and the number of species $N(t)$ is a random process.

References

T. Antal and P. L. Krapivsky. Exact solution of a two-type branching process: clone size distribution in cell division kinetics. *J. Stat. Mech.*, page P07028, 2010.

K. B. Athreya and P. E. Ney. *Branching Processes.* Dover Publications Inc., New York, 2004.

N. Bailey. *The Elements of Stochastic Processes with Applications to the Natural Sciences.* John Wiley & Sons, 1964.

L. C. Evans. *Partial Differential Equations.* AMS, 1998.

R. G. FitzJohn. Quantative traits and diversification. *Syst. Biol.*, 59(6):619–633, 2010.

R. G. FitzJohn. `Diversitree:` comparative phylogenetic analyses of diversification in R. *Meth. Ecol. Evol.*, 3:1084–1092, 2012.

R. G. FitzJohn, W. P. Maddison, and S. P. Otto. Estimating trait–dependent speciation and extinction rates from incompletely resolved phylogenies. *Syst. Biol.*, 58(6):595–611, 2009.

P. Jagers. The proportions of individuals of different kinds in two–type populations. a branching process problem arising in biology. *J. Appl. Prob.*, 6:249–260, 1969.

S. Janson. Functional limit theorems for multitype branching processes and generalized Pólya urns. *Stoch. Proc. Appl.*, 110:177–245, 2004.

W. P. Maddison, P. E. Midford, and S. P. Otto. Estimating a binary character's effect on speciation and extinction. *Syst. Biol.*, 56(5):701–710, 2007.

C. J. Mode. *Multitype Branching Processes Theory and Applications.* American Elsevier Publishing Co. Inc., New York, 1971.

S. Sagitov and M. Serra. Multitype Bienaymé–Galton–Watson processes escapting extinction. *Adv. Appl. Probab.*, 41:225–246, 2009.

A. Y. Yakovlev and N. M. Yanev. Relative frequencies in multitype branching processes. *Ann. Appl. Prob.*, 19(1):1–14, 2009.

A. Y. Yakovlev and N. M. Yanev. Limiting distributions for multitype branching processes. *Stoch. Ann. Appl.*, 28:1040–1060, 2010.
